# Minimally Invasive Breast Biopsy After Neoadjuvant Systemic Treatment to Identify Breast Cancer Patients with Residual Disease for Extended Neoadjuvant Treatment: A New Concept

**DOI:** 10.1245/s10434-023-14551-8

**Published:** 2023-11-10

**Authors:** André Pfob, Lie Cai, Andreas Schneeweiss, Geraldine Rauch, Bettina Thomas, Benedikt Schaefgen, Sherko Kuemmel, Toralf Reimer, Markus Hahn, Marc Thill, Jens-Uwe Blohmer, John Hackmann, Wolfram Malter, Inga Bekes, Kay Friedrichs, Sebastian Wojcinski, Sylvie Joos, Stefan Paepke, Tom Degenhardt, Joachim Rom, Achim Rody, Marion van Mackelenbergh, Maggie Banys-Paluchowski, Regina Große, Mattea Reinisch, Maria Margarete Karsten, Chris Sidey-Gibbons, Markus Wallwiener, Michael Golatta, Joerg Heil

**Affiliations:** 1 Department of Obstetrics and Gynecology, Heidelberg University Hospital, Heidelberg University, Heidelberg, Germany; 2 MD Anderson Center for INSPiRED Cancer Care (Integrated Systems for Patient-Reported Data), The University of Texas MD Anderson Cancer Center, Houston, TX USA; 3 National Center for Tumor Diseases, Heidelberg University Hospital and German Cancer Research Center, Heidelberg, Germany; 4 Institute of Biometry and Clinical Epidemiology, Charité – Universitätsmedizin Berlin, Freie Universität Berlin, Humboldt-Universität zu Berlin, Berlin, Germany; 5 Coordination Centre for Clinical Trials (KKS), University Heidelberg, Heidelberg, Germany; 6 Breast Unit, Kliniken Essen-Mitte, Essen, Germany; 7 Department of Gynecology/Breast Unit, University Hospital Rostock, Rostock, Germany; 8 Department of Gynecology/Breast Unit, University Hospital Tuebingen, Tübingen, Germany; 9 Department of Gynecology and Gynecological Oncology/Breast Unit, Agaplesion Markus Hospital Frankfurt, Frankfurt, Germany; 10 Department of Gynecology with Breast Center, Charité – Universitätsmedizin Berlin, Freie Universität Berlin and Humboldt Universität zu Berlin, Berlin, Germany; 11 Department of Gynecology/Breast Unit, Marienhospital, Witten, Germany; 12 Department of Gynecology and Obstetrics, Medical Faculty, Breast Cancer Center, University of Cologne, Cologne, Germany; 13 Department of Gynecology/Breast Unit, University Hospital Ulm, Ulm, Germany; 14 Department of Gynecology/Breast Unit, Jerusalem Hospital Hamburg, Hamburg, Germany; 15 Department of Gynecology and Obstetrics, Breast Cancer Center, Klinikum Bielefeld Mitte GmbH, Bielefeld, Germany; 16 Radiologische Allianz Hamburg, Hamburg, Germany; 17 Frauenklinik, Interdisziplinäres Brustzentrum des Klinikums rechts der Isar der Technischen Universität München, Munich, Germany; 18 Department of Gynecology/Breast Unit, University Hospital Munich, Munich, Germany; 19 Department of Gynecology/Breast Unit, Klinikum Frankfurt-Höchst, Frankfurt, Germany; 20 Department of Gynecology/Breast Unit, University Hospital Schleswig-Holstein, Lübeck, Germany; 21 Department of Gynecology/Breast Unit, University Hospital Schleswig-Holstein, Kiel, Germany; 22 Department of Gynecology/Breast Unit, University Hospital Halle, Halle, Germany; 23 Department of Symptom Research, The University of Texas MD Anderson Cancer Center, Houston, TX USA; 24 Breast Unit, Klinikum Sankt Elisabeth, Heidelberg, Germany

**Keywords:** Neoadjuvant systemic treatment, Vacuum-assisted biopsy, Residual disease, Pathologic complete response, Extended neoadjuvant treatment

## Abstract

**Background:**

Breast cancer patients with residual disease after neoadjuvant systemic treatment (NAST) have a worse prognosis compared with those achieving a pathologic complete response (pCR). Earlier identification of these patients might allow timely, extended neoadjuvant treatment strategies. We explored the feasibility of a vacuum-assisted biopsy (VAB) after NAST to identify patients with residual disease (ypT+ or ypN+) prior to surgery.

**Methods:**

We used data from a multicenter trial, collected at 21 study sites (NCT02948764). The trial included women with cT1-3, cN0/+ breast cancer undergoing routine post-neoadjuvant imaging (ultrasound, MRI, mammography) and VAB prior to surgery. We compared the findings of VAB and routine imaging with the histopathologic evaluation of the surgical specimen.

**Results:**

Of 398 patients, 34 patients with missing ypN status and 127 patients with luminal tumors were excluded. Among the remaining 237 patients, tumor cells in the VAB indicated a surgical non-pCR in all patients (73/73, positive predictive value [PPV] 100%), whereas PPV of routine imaging after NAST was 56.0% (75/134). Sensitivity of the VAB was 72.3% (73/101), and 74.3% for sensitivity of imaging (75/101).

**Conclusion:**

Residual cancer found in a VAB specimen after NAST always corresponds to non-pCR. Residual cancer assumed on routine imaging after NAST corresponds to actual residual cancer in about half of patients. Response assessment by VAB is not safe for the exclusion of residual cancer. Response assessment by biopsies after NAST may allow studying the new concept of extended neoadjuvant treatment for patients with residual disease in future trials.

**Supplementary Information:**

The online version contains supplementary material available at 10.1245/s10434-023-14551-8.

With an estimated 2.3 million new cases in 2020, breast cancer has become the most commonly diagnosed cancer worldwide.^1^ Neoadjuvant systemic treatment (NAST) is routinely applied to women with locally advanced and/or HER2-positive or triple-negative (TNBC) breast cancer.^2^ Breast cancer patients with residual disease after NAST have a worse prognosis compared with those achieving a pathologic complete response (pCR).^3^ For non-pCR patients with TNBC or HER2-positive disease, post-neoadjuvant treatment with capecitabine or trastuzumab emtansine (T-DM1) showed improved survival.^4,5^

Currently, post-neoadjuvant treatment for non-pCR patients is administered after surgery as histopathologic evaluation of the surgical specimen is required to confirm residual disease. Non-surgical tools such as post-neoadjuvant imaging showed good performance to assess treatment response in general but are inaccurate to definitely assess whether or not residual tumor remains.^6–8^ Identifying patients with residual disease to administer tailored, targeted treatment prior to surgery (i.e., extended neoadjuvant treatment) might be an opportunity to further improve survival for these patients. Previous studies showed the potential of vacuum-assisted biopsies (VABs) after NAST to identify patients with pCR for the omission of surgery.^9,10^ The aim of this study was to explore the feasibility of VAB after NAST to identify patients with HER2-positive breast cancer or TNBC and residual disease (ypT+ or ypN+), who might benefit from tailored, extended neoadjuvant treatment in future trials.

## Methods

This study was performed in line with the principles of the Declaration of Helsinki. Approval was granted by the respective Ethics Committees at all study sites (lead ethics approval Heidelberg University).

### Patient Recruitment

For this secondary analysis, we used data from the RESPONDER trial (NCT02948764), a multicenter, prospective trial that aimed to evaluate the performance of VAB to exclude residual disease after NAST but prior to surgery.^11^ Data were collected at 21 study sites in Germany from 2017 to 2019. Patients aged 18 years or older with cT1-3, cN0-1 breast cancer of any tumor biology with a partial or complete response to NAST as evaluated by post-neoadjuvant imaging were included in the trial. A clip marker by physician choice was placed into the target lesion at the time of diagnosis to mark the original tumor bed. Patients underwent NAST for at least 12 weeks. VAB was performed at the day of surgery or the day before. To standardize response to treatment at the 21 study sites, physicians specialized in breast radiology (either radiologists, or, in Germany, also gynecologists) performed the examinations.

The trial was approved by the respective Institutional Review Board and Ethics Committee. All human participants gave written informed consent.

### Outcomes and Definitions

Assessment of tumor response to NAST was evaluated within the clinical routine using ultrasound and/or MRI by physician choice. Recent meta-analyses suggest equivalent performance of ultrasound and MRI to evaluate response to NAST.^8^ Thus, ultrasound was deemed sufficient to evaluate response to NAST on its own. Additionally, mammography was used to account for potential DCIS. We considered any residual disease on ultrasound and/or or MRI as residual disease on routine imaging, whereas complete response on routine imaging was assumed if ultrasound and/or MRI showed no residual disease and no potential DCIS was seen on mammography.

As previously described,^9,11^ VAB was performed under the guidance of mammography or sonography after the completion of NAST but prior to surgery. At least six specimens with needles sizes from 10 to 7 gauge were taken during the VAB procedure. Histopathologic evaluation of the VAB specimens was categorized as follows: (1) residual tumor cells in the VAB specimen; (2) no residual tumor cells in the VAB specimen, but the VAB specimen was an unclear representative of the former tumor region (no signs of fibrosis); and (3) no residual tumor in the VAB specimen and representative of the former tumor region (signs of fibrosis)

In our present study that aimed to identify patients for extended neoadjuvant treatment, we considered VAB category (1) as tumor positive biopsy, and type (2) and (3) as tumor negative biopsy.

Pathological evaluation of the surgical specimen served as the gold standard for the definition of pCR: pCR was assumed if no residual invasive or in situ tumor cells were found in the breast and axillary lymph nodes (ypT0 and ypN0). The results of imaging and VAB were compared against the results of the surgical specimen. Although breast VAB cannot reflect axillary status directly, we considered residual disease as ypT+ or ypN+ in this study, to keep the comparison consistent with post-neoadjuvant protocols. The positive predictive value (PPV) of VAB was considered the main outcome measure, and additional diagnostic metrics such as specificity, sensitivity, and negative predictive value (NPV) were calculated.

### Statistical Analysis

We performed a descriptive analysis to illustrate the distribution of the baseline characteristics of the pCR and non-pCR cohorts. We used the Chi-square test for categorial data and the *t*-test for continuous data to compare baseline characteristics. A *p*-value <0.05 was considered a significant difference. For the diagnostic performance evaluation, we created confusion matrixes of the binary NAST response assessment of VAB and imaging compared with surgery and then calculated the respective diagnostic metrics. SPSS (IBM Corporation, Armonk, NY, USA) and R version 4.1.1 (The R Foundation for Statistical Computing, Vienna, Austria) were used for all analyses.

## Results

### Patient Selection

**Fig. 1:**
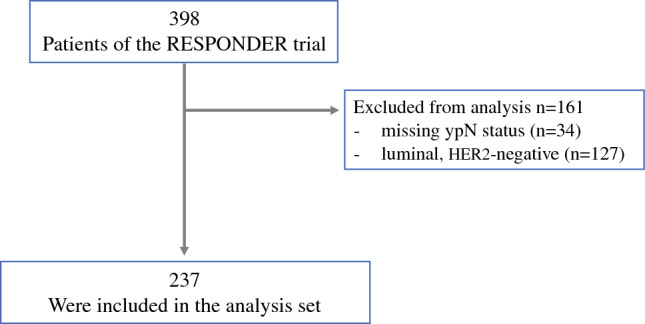
Flowchart of eligible patients used for analysis

Of 398 patients included in the original trial, 161 were excluded due to luminal, HER2-negative tumor biology or missing ypN status. The remaining 237 patients with HER2-positive or TNBC subtype were included in the analysis (Fig. 1).

### Baseline Clinical Characteristics

Of 237 patients, 57.4% (136/237) achieved a pCR. Hormone receptor (HR)-positive, HER2-positive subtype was observed in 15.2% (36/237) of patients, HR negative, HER2-positive in 31.6% (75/237), and TNBC subtype in 53.2% (126/237). Ultrasound was used in 97.9% (232/237) of patients to evaluate response to NAST, whereas MRI was used in 2.1% (5/237). Of all VABs, 183 (77.5%) were ultrasound-guided biopsies and 53 (22.5%) were stereotactic-guided biopsies (clip marker not clearly visible with ultrasound). There were significant differences between the pCR and non-pCR groups with respect to mean age (50.0 vs. 55.9; *p* = 0.003). Further details of the baseline clinical characteristics are shown in Table 1.

**Table 1:** Baseline clinical characteristics comparison between the pCR and non-pCR groups

Characteristics	Whole cohort [*n* = 237]	pCR [*n* = 136]	non- pCR [*n* = 101]	*p*-Value
Age, years (SD)	52.5 (12.5)	50.0 (12.1)	55.9 (12.2)	0.003
Imaging modality after NAST				
Mammography	56 (23.6)			
Ultrasound	232 (97.9)			
MRI	5 (2.1)			
ycT status				< 0.001
ycT0	116 (48.9)	83 (61.0)	33 (32.7)	
ycT+	121 (51.1)	53 (39.0)	68 (67.3)	
cN status				0.987
cN0	166 (71.6)	95 (72.0)	71 (71.0)	
cN+	66 (28.4)	37 (28.0)	29 (29.0)	
Missing	5			
ycN status				0.280
ycN0	185 (89.8)	107 (92.2)	78 (86.7)	
ycN+	21 (10.2)	9 (7.8)	12 (13.3)	
Missing	31			
Grade				0.190
1	1 (0.4)	1 (0.7)	0 (0)	
2	76 (32.9)	37 (27.2)	39 (39.4)	
3	154 (66.7)	95 (69.9)	59 (59.6)	
Missing	6			
Tumor subtype				0.141
HR+, HER2+	36 (15.2)	26 (19.1)	10 (9.9)	
HR+, HER2−	75 (31.6)	40 (29.4)	35 (34.7)	
TNBC	126 (53.2)	70 (51.5)	56 (55.4)	
VAB needle size				0.887
7G	28 (12.3)	18 (13.7)	10 (10.3)	
8G	109 (47.8)	62 (47.3)	47 (48.5)	
9G	11 (4.8)	6 (4.6)	5 (5.2)	
10G	80 (35.1)	45 (34.4)	35 (36.1)	
Missing	7			
VAB guidance				0.491
Sonographic	183 (77.5)	102 (75.6)	81 (80.2)	
Stereotactic	53 (22.5)	33 (24.4)	20 (19.8)	
Tumor response				
pCR	136 (57.4)	–	–	
Non-pCR	101 (42.6)	–	–	

Data are expressed as *n* (%) unless otherwise specified

*pCR* pathologic complete response, *SD* standard deviation, *HER2* human epidermal growth factor receptor 2, *HR* hormone receptor, *TNBC* triple-negative breast cancer, *VAB* vacuum-assisted biopsy, *NAST* neoadjuvant systemic treatment

### Diagnostic Performance of Vacuum-Assisted Biopsy and Routine Imaging for Response Assessment to Neoadjuvant Systemic Treatment

Table 2 shows the diagnostic performance of VAB and routine imaging to detect residual cancer after NAST (ypT+ or ypN+). Tumor cells in the VAB indicated a surgical non-pCR in all patients (73/73; PPV 100%), and no tumor cells in the VAB indicated a surgical pCR in 82.9% of patients (136/164; NPV). PPV and NPV of routine imaging after NAST were 56.0% (75/134) and 74.8% (77/103), respectively. Specificity of VAB was higher (100%, 136/136) compared with that of imaging (56.6%, 77/136). Sensitivity of the VAB was 72.3% (73/101), and 74.3% for sensitivity of imaging (75/101), indicating that residual cancer was missed by VAB in 27.7% of patients and by imaging in 25.7%, respectively, compared with the surgical specimen.

**Table 2:** Diagnostic performance of vacuum-assisted biopsy and imaging to detect residual cancer after neoadjuvant treatment

Diagnostic metric	Vacuum-assisted biopsy	Imaging
Accuracy	88.2% (209/237)	64.1% (152/237)
Sensitivity	72.3% (73/101)	74.3% (75/101)
Specificity	100.0% (136/136)	56.6% (77/136)
PPV	100.0% (73/73)	56.0% (75/134)
NPV	82.9% (136/164)	74.8% (77/103)

*PPV* positive predictive value, *NPV* negative predictive value

Table 3 shows the confusion matrix of VAB and imaging.

**Table 3:** Confusion matrix of VAB and imaging

Whole cohort (*n* = 159)		Surgically confirmed	
		Residual cancer	pCR
VAB	Residual cancer	73	0
	No residual cancer	28	136
Imaging	Residual cancer	75	59
	No residual cancer	26	77

*pCR* pathologic complete response, *VAB* vacuum-assisted biopsy

### Subgroup Analysis

Table 4 shows the diagnostic performance of VAB in the TNBC and HER2-positive subgroups. Diagnostic performance was comparable with a descriptively lower sensitivity in HR-negative, HER2-positive patients (50.0%, 5/10) compared with HR-positive, HER2-positive patients (80.0%, 28/35), and TNBC patients (71.4%, 40/56).

**Table 4:** Diagnostic performance of VAB and imaging in the TNBC and HER2-positive subgroups

Diagnostic metrics	TNBC [*n* = 126]	HER2+, HR− [*n* = 36]	HER2+, HR+ [*n* = 75]
VAB			
Accuracy	87.3% (110/126)	86.1% (31/36)	90.7% (68/75)
Sensitivity	71.4% (40/56)	50.0% (5/10)	80.0% (28/35)
Specificity	100.0% (70/70)	100.0% (26/26)	100% (40/40)
PPV	100.0% (40/40)	100.0% (5/5)	100% (28/28)
NPV	81.4% (70/87)	83.9% (26/31)	85.1% (40/47)
Imaging			
Accuracy	67.5% (85/126)	58.3% (21/36)	61.3% (46/75)
Sensitivity	76.8% (43/56)	80.0% (8/10)	68.6% (24/35)
Specificity	60.0% (42/72)	50.0% (13/26)	55.0% (22/40)
PPV	60.6 (43/71)	38.1% (8/21)	57.1% (24/42)
NPV	76.4 (42/55)	86.7% (13/15)	66.7% (22/33)

*PPV* positive predictive value, *NPV* negative predictive value, *VAB* vacuum-assisted biopsy, *TNBC* triple-negative breast cancer, *HER2* human epidermal growth factor receptor, *HR* hormone receptor

Table 5 shows details on the patients with residual cancer correctly identified by VAB (true positives). Of these 73 patients with residual disease by VAB and surgery, 28.8% (21/73) had ycT0 on routine imaging after NAST.

**Table 5:** Patients with residual cancer correctly identified by VAB

	*N* = 73 (%)
Tumor subtype	
HER2+, HR−	5 (6.8)
HER2+, HR+	28 (38.4)
TNBC	40 (54.8)
Imaging modality used after NAST	
Mammography	13 (17.8)
Ultrasound	71 (97.3)
MRI	2 (2.7)
Results of routine imaging after NAST	
ycT0	21 (28.8)
ycT+	52 (71.2)
ycN status	
ycN0	61 (83.6)
ycN+	6 (8.2)
Missing	6 (8.2)
Histopathologic results	
ypT0, ypN0	0 (0)
ypT+, ypN0	57 (78.1)
ypT0, ypN+	0 (0)
ypT+, ypT+	16 (21.9)
ypT stage	
ypT0	0 (0)
ypT1a	15 (20.5)
ypT1b	13 (17.8)
ypT1c	19 (26.0)
ypT2	14 (19.2)
ypT3	0 (0)
ypTis	12 (16.4)

*HER2* human epidermal growth factor receptor 2, *HR* hormone receptor, *TNBC* triple-negative breast cancer, *NAST* neoadjuvant systemic treatment

## Discussion

In this study, we demonstrate that VAB after NAST is a reliable tool to identify HER2-positive or TNBC patients with residual disease after NAST prior to surgery who might benefit from tailored extended neoadjuvant treatment. PPV of VAB was higher compared with routine imaging after NAST: PPV 100.0% (73/73) versus 56.0% (75/134). Thus, VAB seems more suitable to select patients who might benefit from tailored extended neoadjuvant treatment. Residual cancer found in a VAB specimen after NAST always corresponds to non-pCR; however, residual cancer assumed on routine imaging after NAST corresponds to actual residual cancer in only about half of patients, which would result in many patients who may undergo unnecessary toxic extended therapy. Sensitivity of VAB and imaging were not adequate to reliably exclude residual cancer in the breast or axilla (sensitivity of VAB 72.3% and 74.3% for sensitivity of imaging). Despite comparable sensitivity, VAB seems the preferred method for extended neoadjuvant treatment indication because of the higher PPV compared with imaging (100% vs. 56.0%). Extended neoadjuvant treatment before surgery for patients with residual cancer based on VAB could have advantages over existing post-neoadjuvant strategies after surgery. It may further improve in vivo sensitivity testing to enable targeted therapies for non-responding residual tumor to improve tumor response, and, at best, reach pCR. This could be especially helpful for tailoring treatment for patients with discordant receptor status before and after NAST.

Patients benefit from NAST not only in terms of surgical downstaging (breast-conserving surgery instead of mastectomy); NAST also allows for early in vivo sensitivity testing to anticancer drugs.^12^ Several clinical trials showed improved survival for high-risk patients who undergo escalated post-neoadjuvant treatment. The CREATE-X trial showed 13.7% improved disease-free survival (DFS; 69.8% vs. 56.1%) and improved overall survival (OS; 78.8% vs. 70.3%) with additional capecitabine for TNBC patients with residual disease after NAST.^4^ The KATHERINE trial showed 11.3% improved DFS (88.3% vs. 77.0%) with additional T-DM1 for HER2-positive patients with residual disease after NAST.^5^ The Keynote-522 trial showed 6% improved event-free survival (EFS; 91.3% vs. 85.3%) with additional (neoadjuvant and post-neoadjuvant) pembrolizumab for patients with early TNBC.^13^ The OlympiA trial showed 8.8% improved invasive DFS (iDFS; 85.9% vs. 77.1%) with additional olaparib for patients with HER2-negative breast cancer and BRCA1 or BRCA2 germline pathogenic variants after local and neoadjuvant treatment.^14^ The MonarchE trial showed 3.5% improved iDFS (92.2% vs. 88.7%) with additional abemaciclib to endocrine therapy for patients with HR-positive, HER2-negative disease at high risk of early recurrence after local treatment.^15^ Based on these findings, many more trials have been started to evaluate the role of post-neoadjuvant treatment for patients with residual disease (e.g. trastuzumab deruxtecan [T-DXd, NCT04622319], T-DM1 plus tucatinib [NCT04457596]). However, response assessment to guide post-neoadjuvant treatment is thus far based on the histopathologic evaluation of the surgical specimen. Using VAB after NAST and prior to surgery to prompt extended neoadjuvant treatment could further improve in vivo sensitivity testing for patients with residual disease via administrating tailored/targeted therapies for non-responding residual tumor prior to surgery. A recent meta-analysis showed that the lower the residual cancer burden (RCB) in patients after NAST is, the more favorable is their long-term prognosis: EFS at 10 years was 88% for patients within the RCB-0 class (equivalent to pCR), compared with 80% for RCB-1, 65% for RCB-2, and 45% for RCB-3.^3^ Thus, lowering RCB, at best until reaching pCR, via extended neoadjuvant treatment may be a promising approach to further improve survival for these patients. Although response assessment via VAB does not allow for such a granular distinction as RCB (RCB 0, 1, 2, 3) but rather a binary evaluation (pCR vs. non-pCR), the concept of targeting residual, resisting tumor cells with additional treatment and the advantage of an additional round of in vivo sensitivity testing before surgery, seems worthwhile. To our knowledge, there are no ongoing trials evaluating the concept of extended neoadjuvant treatment, which should thus be considered a conceptual or potential trial design in the future. Some trials are ongoing to identify high-risk patients with a high risk for relapse after standard neoadjuvant chemotherapy to guide escalated or targeted post-neoadjuvant therapy; however, this again refers to post-neoadjuvant treatment after surgery.^16^ Tailored extended neoadjuvant treatment could be especially helpful for tailoring treatment for patients with discordant receptor status before and after NAST (about 20% of patients lose HER2 expression during NAST, 3% acquire HER2 expression, 10% acquire estrogen receptor (ER) expression, and 5% lose ER expression).^17^ Furthermore, future studies should investigate the concordance of immunohistochemistry between the post-neoadjuvant VAB and the surgical specimen. Although we assume that the discrepancy with the post-neoadjuvant VAB is much lower compared with the pre-neoadjuvant biopsy, evidence is scarce.

Another potential advantage of extended neoadjuvant treatment by VAB is time to treatment. A considerable amount of time lays between the completion of NAST and the start of systematic adjuvant treatment. Recent data suggest that the average time interval between the end of NAST and surgery is about 28 days, which is in line with a recommendation to perform surgery about 2–4 weeks after the completion of NAST to give leukocytes time to recover.^18,19^ As for adjuvant treatment after surgery, a National Cancer Database study showed it took, on average, 2–4 weeks to begin adjuvant treatment after surgery.^20^ Thus, patients with residual disease after NAST pass a time of 4–8 weeks without any systemic treatment between the completion of NAST to the beginning of systemic adjuvant treatment, with potential risks of further disease progression. Extended neoadjuvant treatment for patients with residual disease based on VAB might improve systemic tumor control and avoid progression.

As discussed above, escalated post-neoadjuvant treatment is currently based on the histopathologic evaluation of the surgical specimen and administered after surgery. This is because thus far no tool except histopathologic evaluation of the surgical specimen can reliably identify patients with residual disease. A recent meta-analysis summarized the diagnostic accuracy of post-neoadjuvant MRI to assess pCR; pooled specificity to detect residual cancer was 78% (among all patients with pCR, 78% were correctly identified) and pooled sensitivity was 92% (among all patients with residual cancer, 92% were correctly identified), while the corresponding values for ultrasound were 90% and 80%, respectively.^8^ Specificity of MRI and ultrasound is lower compared with that of VAB after NAST (specificity of 100% in our sample), meaning that VAB is more suitable to safely prompt extended neoadjuvant treatment strategies for patients evaluated to have residual cancer; with MRI or ultrasound we would administer unnecessary, toxic systemic treatment to about 20% of patients who actually do not have residual disease (false-positives). However, the lower sensitivity of VAB means that some patients with residual cancer, who might be eligible for extended-neoadjuvant treatment, will be missed and will continue to undergo standard of care post-neoadjuvant treatment.

**Fig. 2:**
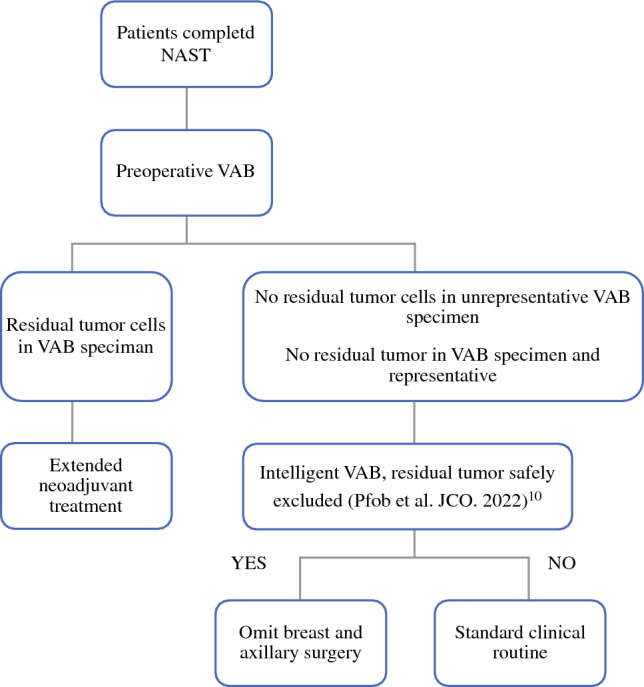
Concept of individualized post-neoadjuvant treatment strategy based on response assessment via vacuum-assisted biopsy. *NAST* neoadjuvant systemic treatment, *VAB* vacuum-assisted biopsy

Performing VAB to evaluate response to NAST prior to surgery cannot only be used to prompt targeted extended neoadjuvant treatment for patients with residual disease but also to de-escalate treatment for patients without residual disease.^21^ The low sensitivity (high rate of missed residual cancer) of VAB alone is in line with our previous research, which showed that for the reliable exclusion of residual cancer, VAB should be combined with imaging and specific patient selection criteria.^9,22^ Recent studies showed that an ‘intelligent VAB’, a machine learning algorithm analyzing VAB variables alongside clinical and patient information, can reliably exclude residual cancer after NAST; these patients without residual disease might be spared breast and axillary surgery.^10,23^ Thus, future trials may evaluate oncologic outcomes of response assessment to NAST via VAB within three patient groups (Fig. 2): (1) patients with residual disease in the conventional VAB specimen who will then receive tailored and targeted extended neoadjuvant treatment before surgery; (2) patients with no residual disease as evaluated by the intelligent VAB who will then omit breast and axillary surgery’ and (3) patients without residual disease in the conventional VAB specimen but for whom residual disease cannot safely be excluded by the intelligent VAB, who will then undergo surgery and standard post-neoadjuvant treatment based on the pathologic evaluation of the surgical specimen. Future studies may also investigate whether (intelligent) VAB cannot only reliably exclude residual disease after NAST but also during the course of NAST to potentially end NAST ahead of schedule.

Our study has some limitations. First, this was a secondary analysis of a previously reported clinical trial.^10^ Second, the sample size was moderate, especially for HER2-positive patients. Larger prospective data are required to validate our findings. Third, trial inclusion criteria specified that a clip marker or target lesion must be visible for the biopsy; patients with dislocated markers were excluded. This may introduce uncertain bias to some degree. Forth, ultrasound, in general, is an operator- and device-dependent modality. To standardize response to treatment at the 21 study sites, physicians specialized in breast radiology performed the examinations. Fifth, this was a diagnostic study focusing on diagnosing pathologic response to NAST but without findings on oncologic outcomes. Recently, the importance of pCR as a surrogate parameter for survival has come under scrutiny, with some trials suggesting that long-term benefits (especially of immune response) may not be reflected by pCR.^24,25^ Thus, the implication of our findings on oncologic outcomes may be evaluated in future clinical trials.

## Conclusion

Minimally invasive biopsies after NAST can identify HER2-positive or TNBC patients with residual disease after NAST prior to surgery and might be more suitable to prompt extended neoadjuvant treatment for these patients than imaging. Based on the response assessment of NAST via minimally invasive biopsies, future trials may evaluate (1) extended neoadjuvant treatment for patients with residual disease, and (2) de-escalated treatment for patients without residual disease, i.e. omission of breast and axillary surgery and, potentially, omission of further chemotherapy. This newly proposed concept of individualized response monitoring to NAST will have to be evaluated in future trials to assess oncologic outcomes.

### Supplementary Information

Below is the link to the electronic supplementary material.

Supplementary file 1 (DOCX 35 KB)

## Data Availability

*Will individual participant data be available (including data dictionaries)?* Yes. *What data in particular will be shared?* Individual participant data that underlie the results reported in this article, after de-identification (text, tables, figures, and appendices). *What other documents will be available?* Study protocol. *When will data be available (start and end dates)?* Immediately following publication. No end date. *With whom?* Researchers who provide a methodologically sound proposal. *For what types of analyses?* To achieve aims in the approved proposal. *By what mechanism will data be made available?* Proposals should be directed to joerg.heil@med.uni-heidelberg.de. To gain access, data requestors will need to sign a data access agreement.
